# *Bacillus subtilis* Early Colonization of *Arabidopsis thaliana* Roots Involves Multiple Chemotaxis Receptors

**DOI:** 10.1128/mBio.01664-16

**Published:** 2016-11-29

**Authors:** Rosalie Allard-Massicotte, Laurence Tessier, Frédéric Lécuyer, Venkatachalam Lakshmanan, Jean-François Lucier, Daniel Garneau, Larissa Caudwell, Hera Vlamakis, Harsh P. Bais, Pascale B. Beauregard

**Affiliations:** a Département de Biologie, Faculté des Sciences, Université de Sherbrooke, Sherbrooke, Québec, Canada; b Department of Plant and Soil Sciences, Delaware Biotechnology Institute, University of Delaware, Newark, Delaware, USA; c Department of Microbiology and Immunobiology, Harvard Medical School, Boston, Massachusetts, USA

## Abstract

Colonization of plant roots by *Bacillus subtilis* is mutually beneficial to plants and bacteria. Plants can secrete up to 30% of their fixed carbon via root exudates, thereby feeding the bacteria, and in return the associated *B. subtilis* bacteria provide the plant with many growth-promoting traits. Formation of a biofilm on the root by matrix-producing *B. subtilis* is a well-established requirement for long-term colonization. However, we observed that cells start forming a biofilm only several hours after motile cells first settle on the plant. We also found that intact chemotaxis machinery is required for early root colonization by *B. subtilis* and for plant protection. *Arabidopsis thaliana* root exudates attract *B. subtilis in vitro*, an activity mediated by the two characterized chemoreceptors, McpB and McpC, as well as by the orphan receptor TlpC. Nonetheless, bacteria lacking these chemoreceptors are still able to colonize the root, suggesting that other chemoreceptors might also play a role in this process. These observations suggest that *A. thaliana* actively recruits *B. subtilis* through root-secreted molecules, and our results stress the important roles of *B. subtilis* chemoreceptors for efficient colonization of plants in natural environments. These results demonstrate a remarkable strategy adapted by beneficial rhizobacteria to utilize carbon-rich root exudates, which may facilitate rhizobacterial colonization and a mutualistic association with the host.

## IMPORTANCE

*Bacillus subtilis* is a plant growth-promoting rhizobacterium that establishes robust interactions with roots. Many studies have now demonstrated that biofilm formation is required for long-term colonization. However, we observed that motile *B. subtilis* mediates the first contact with the roots. These cells differentiate into biofilm-producing cells only several hours after the bacteria first contact the root. Our study reveals that intact chemotaxis machinery is required for the bacteria to reach the root. Many, if not all, of the *B. subtilis* 10 chemoreceptors are involved in the interaction with the plant. These observations stress the importance of root-bacterium interactions in the *B. subtilis* lifestyle.

## INTRODUCTION

In nature, bacteria face ever-changing environmental conditions, which call for rapid and constant adaptations to ensure optimal growth and proliferation. Motility is an important survival strategy used by bacteria to move toward increasing gradients of attracting molecules or away from repellent molecules. This process, known as chemotaxis, allows organisms to reach ecological niches with higher nutrient concentrations while avoiding toxins. Bacterial chemotaxis is triggered when a stimulating molecule binds to its cognate chemoreceptor, causing downstream modification of the CheA kinase phosphorylation state and of its response regulator, CheY. CheY then interacts with the flagellum motor to affect the motile behavior of the bacterium (reviewed in references 1 and 2). The core proteins of the chemotaxis signaling pathway, CheA and CheY, are found throughout *Archaea* and Gram-negative and Gram-positive bacteria, suggesting that this cellular process is very ancient and well conserved (1, 3).

The abundance of chemoreceptors encoded by bacterial genomes appears to be more correlated with the lifestyles of individual species rather than with their genome sizes. Bacteria whose genome contains many chemoreceptor genes typically possess complex behaviors, such as cell differentiation or an ability to establish relationships with other living organisms (4). Chemotaxis has a major role for various plant-associated bacteria, whether they are beneficial or pathogenic. *Rhizobium leguminosarum*, *Pseudomonas fluorescens*, *Azotobacter chroococcum*, *Sinorhizobium meliloti*, and many others are attracted by root exudates (5–9). For some of these, chemotaxis toward plant-secreted molecules is also required for establishing the initial host-microbe interaction (7, 10–15). Similarly, the causative agents of certain plant diseases, such as *Ralstonia solanacearum* and *Dickeya dadantii* 3937, necessitate directed motility mediated by chemotaxis for their plant virulence activity (16–18).

While the first studies on chemotaxis were done on the enteric bacteria *Escherichia coli* and *Salmonella enterica* serovar Typhimurium, this process has also been examined in the Gram-positive organism *Bacillus subtilis*. This bacterium’s chemotaxis system is more complex and closer to that of *Archaea* than the machinery of enteric bacteria (1). Among the 10 chemoreceptors encoded by the *B. subtilis* genome, several are well characterized and possess known ligands, among which we find amino acids, various carbon sources, and oxygen (19–21). Interestingly, *B. subtilis* shows chemotaxis toward a broad variety of amino acids with no preference for those of high nutritional value, suggesting that chemotaxis could help locate favorable environmental niches such as plant roots (22). However, the importance of the various chemoreceptors of *B. subtilis* in a naturally relevant context, such as plant root colonization, has not been examined.

*B. subtilis* is a well-known soil-dwelling bacterium that can be found in interaction with the roots of various plants (23–25). This interaction is beneficial for both concerned parties, as *B. subtilis* possesses many activities that promote the growth and health of plants. Concomitantly, plants secrete large amounts of carbon sources in otherwise relatively poor soil (26, 27). *B. subtilis* root colonization persisting for at least 24 h requires the formation of a biofilm, which is induced by plant-produced molecules, such as cell wall polysaccharides (28) and malic acid (29, 30). Bacterial biofilms are characterized by the formation of a multicellular bacterial community encompassed by a self-secreted matrix. In *B. subtilis*, only a subset of cells present in the biofilm secrete the matrix, a cellular function that is incompatible with cell motility, making both processes mutually exclusive (31). When looking at the timing of *B. subtilis* biofilm formation on the root, there is almost no activity of matrix-associated gene promoters when the cells initially contact the root (28). This observation thus suggests that another cell type is involved in the early steps of root colonization.

Here, we used an *in vitro Arabidopsis thaliana* root colonization assay to evaluate the importance of motility and chemotaxis for the successful establishment of a *B. subtilis* population on plant roots. We observed that early colonization of *Arabidopsis thaliana* by wild-type (WT) *B. subtilis* required the bacteria to swim and be able to chemotax. On the plant roots, *B. subtilis* differentiation from motile cells into biofilm-producing cells happened only 4 to 8 h after the first contact, which is consistent with the fact that biofilm formation has been shown to be required for persistent long-term colonization. Interestingly, many chemoreceptors are involved in the early establishment of a *B. subtilis* population on the root, suggesting that the plant secretes a variety of molecules serving as attractants for the bacterium.

## RESULTS

### Motility and chemotaxis are required for root colonization of *A. thaliana.*

**FIG 1:**
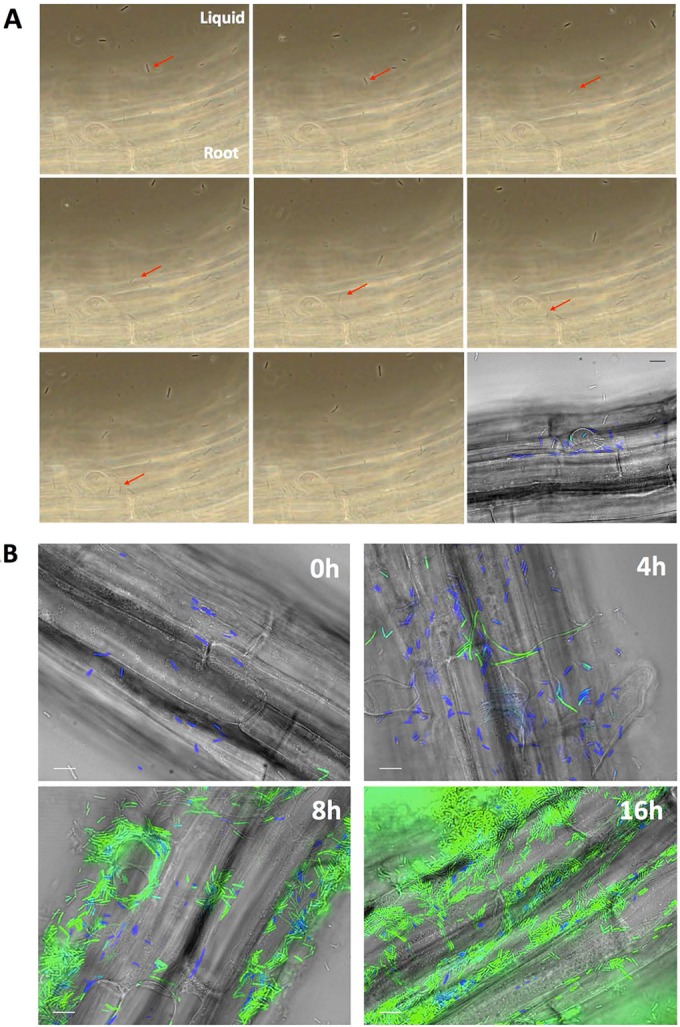
(A) Sequential phase-contrast pictures of an *A. thaliana* root inoculated with *B. subtilis* NCIB 3610 cells harboring *P*_*tapA*_-*yfp* and *P*_*hag*_-*cfp* reporters. The medium used was MSNg, and imaging started immediately after the inoculation. The red arrow points to a cell swimming toward the root and settling on it. Magnification, ×60. Each picture is separated by 0.5 s; the complete movie can be found in Movie S1 in the supplemental material. The last image is a fluorescence picture of the same frame with overlays of fluorescence (false-colored green for YFP and blue for CFP) and transmitted light (gray) images. (B) When *B. subtilis* cells colonize *A. thaliana* roots, they first express motility genes, followed by matrix genes. NCIB3610 cells harboring P_*tapA*_-*yfp* and P_*hag*_-*cfp* were coincubated with *A. thaliana* seedlings and imaged at 0, 4, 8, and 16 h postinoculation. Shown are overlays of fluorescence (false-colored green for YFP and blue for CFP) and transmitted light (gray) images. Pictures are representative of at least 12 independent roots. Bars, 10 μm.

Recently, we and others demonstrated that biofilm formation is required for *B. subtilis* to colonize *A. thaliana* roots after a period of 24 h or longer (28, 32). To better understand the beginning of root colonization, we filmed the first contact between bacteria and the plant. A dual-fluorescent reporter *B. subtilis* strain was used to examine the expression of the motility and biofilm cellular machineries during the initiation of colonization. Specifically, this strain carries a *cfp* gene placed under the control of the flagellar *hag* gene promoter (*P_hag_-cfp*) and a *yfp* gene placed under the control of the biofilm matrix *tapA* gene promoter (*P_tapA_-yfp*) (31). Real-time bright-field microscopy imaging of a *A. thaliana* Col-0 seedling inoculated with this *B. subtilis* strain was initiated. We observed that most bacteria demonstrated swimming motility, and several of them settled on the root (Fig. 1A; see also Movie S1 in the supplemental material). Fluorescent imaging confirmed that the vast majority of cells adhering to the root were expressing cyan fluorescent protein (CFP) and thus were actively transcribing motility-related genes (Fig. 1A, bottom right image, and B). Since motility and biofilm formation are two mutually exclusive cellular processes in *B. subtilis* (31), we also noted that only a very limited number of cells expressed *P_tapA_-yfp* in the first moments of interaction with the root. Using the same dual-reporter strain, we examined the early time points of root colonization. Confirming our previous observation, we established that the initial *B. subtilis* population on the roots at 0 and 4 h postinoculation expressed *P_hag_-cfp*. Several *P*_*tapA*_*-yfp*-expressing cells showing the elongated morphology typical of matrix-producing cells were also present on the root by 4 h postinoculation (Fig. 1B). At 8 h postinoculation, most of the *B. subtilis* cells present on the root expressed *P_tapA_-yfp*, and this reporter was also strongly expressed at later time points (Fig. 1B) (28). These observations suggest that while motile *B. subtilis* cells first interact with the roots, they differentiate into matrix-producing cells to start forming a biofilm between 4 and 8 h postinoculation.

**FIG 2:**
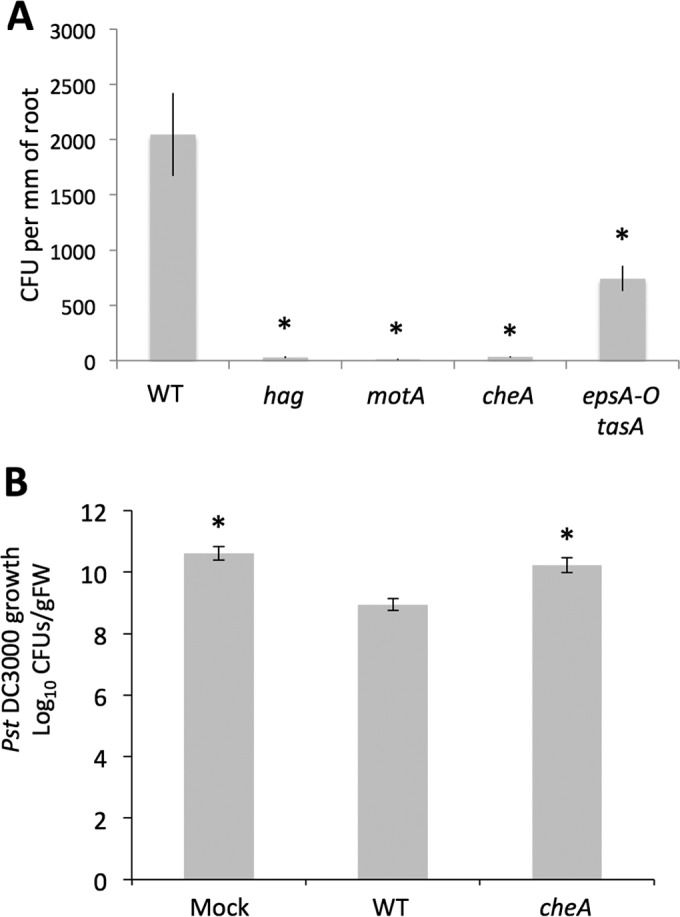
(A) Quantification of root colonization by *B. subtilis* and various mutants 4 h postinoculation. One-week-old *A. thaliana* seedlings were coincubated with either WT or mutant *B. subtilis* strains in MSNg. After 4 h of incubation, the roots were collected, measured, washed in PBS, and sonicated to disperse the bacteria. CFU were evaluated after overnight culture on LB agar. For each strain, the bar represents the mean and standard deviation of four biological replicates. (B) Protection of *A. thaliana* against *P. syringae* pv*. tomato* DC3000 conferred by WT *B. subtilis* or a chemotaxis mutant. Three-week-old *A. thaliana* Col-0 plants were rhizo-inoculated with a mock control, WT NCIB3610 *B. subtilis*, or a *cheA* mutant and infiltrated with strain DC3000. After 72 h of infection, strain DC3000 growth in leaves was quantified by CFU counts. For each strain, the bar represents the mean and standard deviation of at least nine biological replicates. Bars marked with an asterisk indicate a significant difference from the WT result.

Because the cells involved in the first stage of root colonization express motility genes, this strongly suggests that swimming is required for early root adherence. To examine this hypothesis, we developed a simple colonization assay allowing us to quantify the number of *B. subtilis* cells attached to the root at 4 h postinoculation. Fluorescence imaging showed that between inoculation and 4 h, biofilm formation was not initiated (Fig. 1B), making it possible to completely detach cells from the root by sonication and to quantify them by CFU counting. Using this assay, we observed that wild-type cells colonized the root at a density of approximately 2,000 CFU per mm of root (Fig. 2A). We then evaluated the capacity of a flagellar mutant (*hag*) and of a flagellar motor mutant (*motA*) to colonize the root. As seen in Fig. 2A, neither of these mutants was able to colonize the root after 4 h, demonstrating that flagellar motility is required for the colonization process. Importantly, this lack of colonization by motility-deficient cells was not due to cell growth impairment, since growth of the strains used in this experiment was identical to wild-type cell growth (see Fig. S1). Since swimming motility and chemotaxis are often linked in bacteria, we then tested whether root colonization could also involve chemotaxis. We used a *cheA B. subtilis* mutant that is incapable of chemotaxis and displays a tumbling phenotype (33). As shown in Fig. 2A, this mutant did not associate with roots 4 h postinoculation. Also, a double mutant deficient for production of the biofilm matrix (*eps tasA*) was still able to colonize the root to some extent (approximately 36%). These results strongly suggest that the first association between *B. subtilis* and *A. thaliana* roots is the result of the bacterial chemoattraction toward the root, which is mediated by plant-secreted molecules. Importantly, *cheA* mutant cells were not impaired in biofilm formation *in vitro* (Fig. S2A), and while they were completely impaired in colonization after 4 h, they were able to colonize the root to some extent (approximately 65%) 16 h postinoculation (Fig. S2B and C). This late colonization most likely results from random contacts between the mutant *cheA* cells and the roots, since given enough time these cells will multiply and swim in every direction. These results reinforce the idea that the absence of colonization by the *cheA* cells at 4 h postinoculation is due to a chemotaxis defect and not a biofilm-forming defect. Of note, *hag* and *motA* mutants showed delayed pellicle formation and a complete defect in root colonization (Fig. S2A to C), which was not unexpected since these strains combine the absence of swimming motility with partially impaired biofilm formation (34, 35).

This attraction toward the root also appears to constitute the first step in the plant protection mechanism provided by *B. subtilis*. Indeed, *B. subtilis* can trigger induced systemic resistance (ISR) in *A. thaliana*, which lowers disease severity of the pathogen *Pseudomonas syringae* pv*. tomato* DC3000. This effect results in lower strain DC3000 CFU counts on the leaves of plants with root-associated *B. subtilis* compared to noninoculated plants, suggesting possible induction of ISR against the aerial pathogen infection (Fig. 2B, compare the mock and WT samples) (36). However, when root inoculation was performed with a *B. subtilis cheA* mutant instead of the WT, we observed that the protection effect was significantly reduced (Fig. 2B). This result suggests that chemotaxis is required for *B. subtilis* association with the root and subsequent plant protection via induction of ISR.

### *A. thaliana* root exudates attract *B. subtilis.*

Since early colonization of *A. thaliana* roots by *B. subtilis* requires chemotaxis, this suggests that under our growth conditions *A. thaliana* secretes one or many molecules serving as attractants. Accordingly, we tested *in vitro* whether *A. thaliana* root exudates attracted *B. subtilis*. The exudates were prepared by incubating *A. thaliana* seedlings in MSN medium (see Materials and Methods for medium composition) for 1 to 2 weeks, after which time the medium was harvested and filtered. The capacity of these exudates to attract *B. subtilis* was then tested using a capillary assay. As seen in Fig. S3, root exudates (RE) attracted many bacteria, and the strength of this attraction increased as the exudate concentrations got higher. The negative control used in this experiment was either water or the medium used to prepare exudates (MSN), both of which had no effect on the cells. This experiment confirmed the chemoattraction activities of *A. thaliana* root exudates.

**FIG 3:**
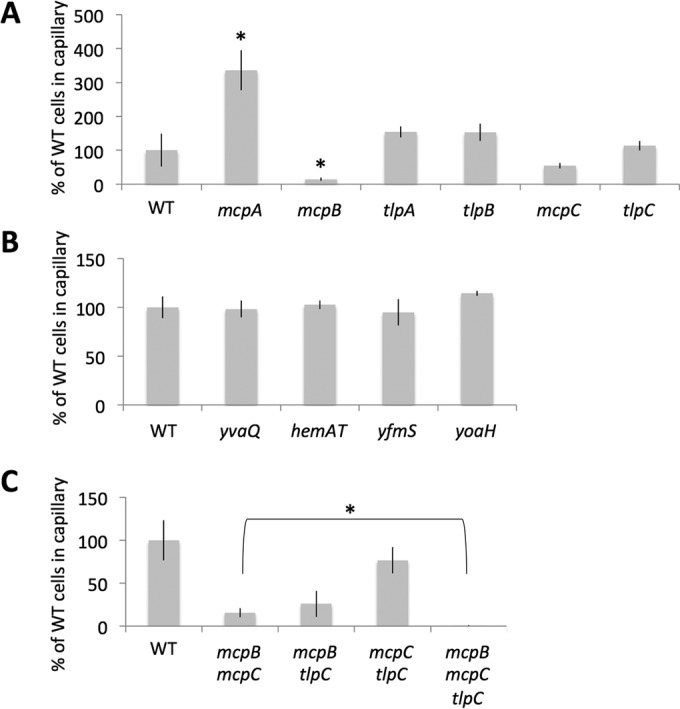
Chemotaxis toward root exudates by various chemoreceptor deletion mutants. Attraction of various *B. subtilis* strains toward *A. thaliana* root exudates was measured via a capillary assay (see Materials and Methods). After incubation, the number of CFU in the capillaries was evaluated from an overnight culture on LB agar; numbers are relative to the number of CFU for the WT *B. subtilis* strain. (A and B) Results for single-deletion mutants; (C) results for combinatorial mutants. For each strain, the bar represents the mean and standard deviation of three biological replicates. In panel A, bars marked with an asterisk were significantly different from the WT result; in panel C, bars marked with an asterisk indicate that results for the mutant strains differed significantly from each other.

We then constructed single-deletion mutants for all 10 *B. subtilis* chemoreceptors and evaluated the capacity of these mutants to respond to root exudates in comparison with WT cells (Fig. 3A and B). Interestingly, more than one chemoreceptor appeared to be involved in response to root exudates. The strain lacking the McpA chemoreceptor consistently showed twice as much attraction toward exudates than WT cells. This observation suggests that McpA could respond to a repellent molecule present in the exudates, the effect of which would be masked by the presence of one or more attracting molecules. Deletion of the McpB chemoreceptor also significantly impacted the attraction toward root exudates. Cells devoid of this chemoreceptor were much less attracted to root exudates than WT cells. While not statistically significant, deletion of *mcpC* also consistently showed a decreased attraction level toward *A. thaliana* root exudates in the various biological replicates. These two results agree well with the existing literature, since McpB and McpC are known to respond to the presence of amino acids and sugars, molecules that are present in root exudates (21, 37–39). None of the other single deletions of chemoreceptors, either characterized or orphan, displayed an effect on the chemoattraction of the strains toward root exudates.

In an attempt to obtain a *B. subtilis* strain completely unresponsive to *A. thaliana* exudates, we evaluated the attraction of the *mcpB mcpC* double mutant. As shown in Fig. 3C, the *mcpB mcpC* mutant displayed about 15% of the WT level of attraction toward exudates, which is an important decrease but also means that the cells are not completely unresponsive. This observation suggests that an additional chemoreceptor(s) could still mediate a small response to the exudate. Many combinatorial mutants were produced and tested (data not shown), and we were able to observe that TlpC was also involved in this response. By itself or in combination with single *mcpB* or *mcpC* deletion (Fig. 3A and C), the *tlpC* mutation did not have an important effect on the attraction response. However, the combination of these three mutations gave rise to cells completely unable to respond to root exudates. This result demonstrated that TlpC mediates a small but reproducible response to root exudates that is independent from the response mediated by the two other receptors. Importantly, the absence of attraction to root exudates of this triple mutant is not due to a general malfunction of the chemotaxis machinery, since the *mcpB mcpC tlpC* mutant could chemotax toward 1% yeast extract, while a *cheA* mutant could not (Fig. S4). This phenotype also cannot be attributed solely to an important swimming bias, since the triple mutant showed a small but not statistically significant reduction of tumbling rate compared to WT cells (Fig. S5). Similarly, the *mcpA* deletion mutant did not display a swimming bias (Fig. S5), strongly suggesting that this deletion would make the cells insensitive to the presence of a repellent present in root exudates, thus causing the increased attraction toward root exudates we observed. In conclusion, these experiments showed that the main chemoreceptors for *A. thaliana* seedling exudates are McpB and McpC, with TlpC playing a minor role in the chemotaxis response.

### Early colonization of *A. thaliana* roots involves multiple chemoreceptors.

**FIG 4:**
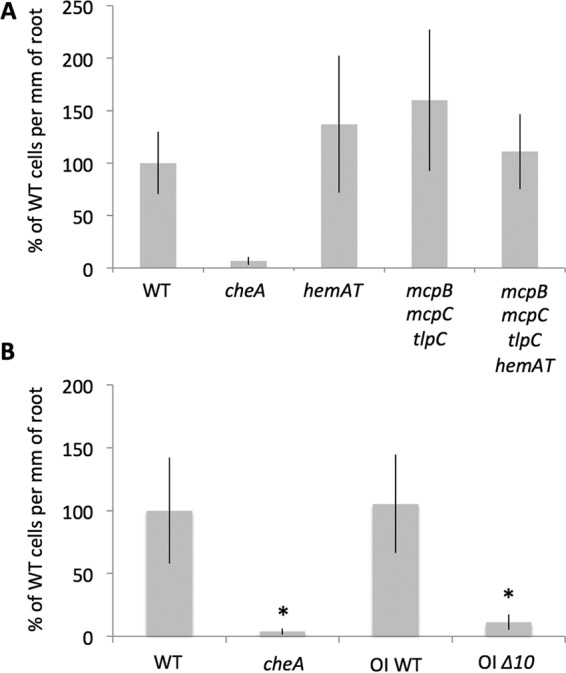
Root colonization assay with chemoreceptor deletion mutants. One-week-old *A. thaliana* seedlings were coincubated with either WT or mutant *B. subtilis* strains in MSNg. After 4 h, roots were collected, measured, washed in PBS, and sonicated to disperse the bacteria. CFU were evaluated after overnight culture on LB agar, and numbers are reported relative to the CFU per millimeter of root for WT *B. subtilis*. For each strain, the bar represents the mean and standard deviation of four biological replicates; bars marked with a asterisk indicate the result differed significantly from that for the WT.

Once we identified the chemoreceptors responding to the molecules present in exudates, we evaluated their roles in early root colonization. The triple *mcpB mcpC tlpC* deletion mutant was inoculated on *A. thaliana* seedlings and incubated for 4 h, after which the number of *B. subtilis* cells present on the root was evaluated. Interestingly, cells with the triple chemoreceptor deletion were still able to colonize the root to a level similar to WT cells (Fig. 4A). *B. subtilis* is capable of aerotaxis, a behavior mediated by the soluble chemoreceptor HemAT, which acts as an oxygen sensor (20). A recent study showed that *hemAT* mutants were outcompeted by wild-type cells during biofilm formation in liquid medium, suggesting that oxygen sensing provides an advantage during the formation of a pellicle at the air-liquid interface (34). Since *B. subtilis* forms a biofilm on *A. thaliana* roots, we investigated if the root colonization capacity of the *mcpB mcpC tlpC* mutant could be mediated by HemAT-mediated oxygen sensing. As shown in Fig. 4A, HemAT had no influence on root colonization, since the single *hemAT* deletion showed colonization levels similar to WT and the quadruple *mcpB mcpC tlpC hemAT* deletion mutant behaved like the *mcpB mcpC tlpC* mutant. Since the biofilm formed on the root is submerged and not at the interface with air, that could explain why aerotaxis does not play a role in root colonization.

To evaluate whether early root colonization by *B. subtilis* is solely mediated by chemoreceptors, we tested the root colonization capacity of a strain in which all 10 genes encoding chemoreceptors were deleted (OI *Δ10*). This strain has a slightly different genetic background than NCIB 3610, so we also examined the colonization capacity of its parental strain (OI WT). As shown in Fig. 4B, both WT strains showed high levels of root colonization, while the *Δ10* strain acted very similarly to the *cheA* mutant. These results indicated that indeed, early root colonization is fully mediated by chemoattraction and chemoreceptors and that a receptor(s) other than McpB, McpC, and TlpC is involved. In an effort to identify the other receptors involved, we produced strains containing the triple deletion *mcpB mcpC tlpC* mutant in combination with the deletion of all the orphan chemoreceptors, either one at a time (YoaH, YvaQ, YfmS) or with the deletion of the McpB TlpA McpA TlpB genetic cluster. Subsequent testing of the root colonization capacities of these strains demonstrated that none of them could recapitulate the *cheA* phenotype (data not shown). To evaluate how important root exudate sensing is in colonizing *A. thaliana* roots, a strain containing a deletion for all chemoreceptors except McpB, McpC, TlpC, and the oxygen-sensing HemAT was constructed; of note, in this construction *mcpB* is under the control of an inducible promoter. As shown in Fig. S6, this strain was still able to colonize the root, indicating that root exudates are sufficient to promote plant colonization. However, since colonization by this mutant was generally less efficient than colonization by WT cells, we propose that other signals, such as unstable molecules, can favor this process.

### Conservation of chemoreceptors in plant growth-promoting *Bacillus* spp.

**FIG 5:**
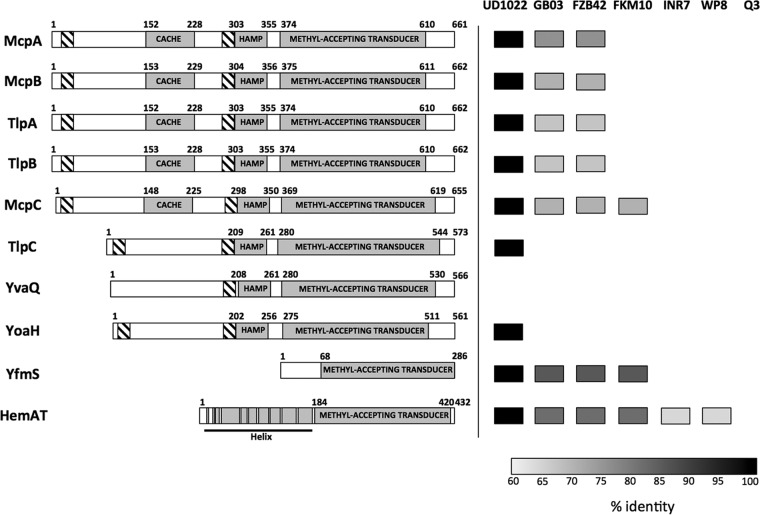
*B. subtilis* NCIB3610 chemoreceptors and conservation in other PGPR *Bacillus* spp. Predicted domains of *B. subtilis* chemoreceptors, according to information on the UniProt website (http://www.uniprot.org), are depicted. Hatched boxes represent the transmembrane domains, while other domains are shown in gray; CACHE domains are predicted to have a role in small-molecule recognition; HAMP domains are composed of an α-helix forming a coiled-coil frequently found in signaling proteins, and methyl-accepting transducer domains are the signaling domains of chemoreceptors. On the right is a depiction of the full-length conservation of each chemoreceptor in different plant-colonizing *Bacillus* strains, namely, *B. subtilis* UD1022 (40), *B. amyloliquefaciens* GB03 and FZB42 (41, 42), *B. methylotrophicus* FKM10 (43), *B. pumilus* INR7 and WP8 (44, 45), and *B. megaterium* Q3 (46).

As depicted in Fig. 5, 7 of the 10 *B. subtilis* chemoreceptors possess two transmembrane domains, 5 of which (McpA, McpB, McpC, TlpA, and TlpB) display a large extracellular portion with a small-molecule recognition CACHE domain. The two others, TlpC and YoaH, have smaller extracellular domains. According to these structures, it appears that the chemoreceptors involved in sensing root exudates (McpB, McpC, and TlpC) all possess a large extracellular domain, which is expected for receptors sensing environmental molecules.

Since *B. subtilis* strain 3610 was shown to strongly colonize the root, we wanted to examine the degree of conservation of these chemoreceptors in other root-colonizing *Bacillus* strains, specifically *B. subtilis* UD1022 (40), *B. amyloliquefaciens* GB03 and FZB42 (41, 42), *B. methylotrophicus* FKM10 (43), *B. pumilus* INR7 and WP8 (44, 45), and *B. megaterium* Q3 (46). The published genomes of these bacterial strains were searched for homologs of all 10 *B. subtilis* chemoreceptors. To ensure high specificity, only receptors with higher than 60% identity for the full-length protein were considered likely homologs. As shown in Fig. 5B, *B. subtilis* plant growth-promoting strain UD1022 showed nearly identical homologs with 9 out of 10 *B. subtilis* chemoreceptors, suggesting that chemotaxis toward root exudates might have very similar roles in root colonization for both of these strains. Interestingly, the *B. amyloliquefaciens* root-colonizing strains displayed a much lower degree of conservation of the chemoreceptors. The root exudate-sensing McpB and McpC proteins were well conserved between strains, both of them showing sequence identity higher than 75%. However, among the three missing receptors in *B. amyloliquefaciens* we found TlpC, shown here to sense a molecule(s) present in *A. thaliana* root exudates. Additionally, Fig. 5 shows the poor conservation of *B. subtilis* 3610 chemoreceptors in the plant-colonizing *B. methylotrophicus*, *B. pumilus*, and *B. megaterium* strains. While *B. pumilus* does not possess homologs of the *B. subtilis* chemoreceptors with the extracellular domain, it was shown to be attracted to rice root exudates (47). Thus, this species most likely encodes a different set of chemoreceptors sensitive to root exudate molecules. These observations suggest that in *Bacillus*, chemoreceptor conservation depends more on genetic relatedness than on responses to environmental stimuli.

## DISCUSSION

A large amount of work has been done on the *B. subtilis* chemotaxis machinery, but its role in a more environmentally relevant context has been lacking so far. Here we show that motile cells, but not biofilm-forming cells, mediate the first contact with *A. thaliana* roots. We demonstrated that cells require a functioning chemotaxis signaling pathway to colonize the root 4 to 8 h postinoculation, and to help a plant’s self-defense reaction against a *P. syringae* pv*. tomato* DC3000 infection. It was previously reported that exudates from rice plants attract *Bacillus* spp. strain*.* 709 and an endophytic *B. pumilus*, while soybean root and seed exudates can attract *B. megaterium* and *B. amyloliquefaciens* (47–49). Moreover, maize root exudates were shown to induce the expression of motility genes in *B. amyloliquefaciens* FZB42, a potent root colonizer (50). Together with these observations, our results make a strong case for root exudates playing an important role in recruiting *B. subtilis* and promoting the colonization of plant roots.

According to our results and the literature, we propose the following colonization model. *B. subtilis* is attracted to the root by plant-secreted molecules, and the first contact with the root surface is dependent on the bacterium’s capacity to move toward the plant. The primary adhesion would likely be reversible, since we detected *eps tasA* mutants that did not produce extracellular matrix on the root after 4 h of incubation (Fig. 2), but observations at later time points revealed that this double mutant eventually detached completely from *A. thaliana* roots (28). Five to six hours following this first contact, *B. subtilis* cells start secreting surfactin (51) and then differentiate into matrix-producing cells, the most abundant cell type on the root at 8 h (Fig. 1B). These observations are consistent with surfactin production preceding matrix gene expression under many conditions (52, 53). Secretion of surfactin and of the biofilm matrix exopolysaccharides and TasA-TapA proteins would then promote a robust and long-lasting attachment of the cells to the root (28, 32, 54).

Using a capillary assay, we observed that McpB, McpC, and TlpC chemoreceptors mediated *B. subtilis* attraction toward *A. thaliana* seedling exudates. McpB detects a subset of amino acids, including asparagine (for which it is the sole receptor), aspartate, glutamine, and histidine (19). McpC mediates taxis toward 17 amino acids as well as numerous sugars and sugar alcohols via an interaction with the phosphotransferase system (PTS) (21, 38). Since root exudates contain various amino acids, sugars, and small acids, our observation that McpB and McpC are the main chemoreceptors active in recognizing *A. thaliana* exudates is consistent with their known activities (39). Although minor, TlpC also plays a role in sensing root exudate, which is the first time this protein has been shown to be involved in a specific chemotaxis *per se* (37). Identification of the molecule(s) sensed by TlpC will be the object of future investigations, and these investigations will give us important clues on the signaling molecules secreted by the plants. We speculate that McpB and McpC mediate general attraction toward roots by binding to common ligands such as amino acids and sugars (19, 21, 38), while TlpC may mediate chemoattraction via specific root-secreted molecules. This hypothesis could be examined by introducing TlpC in a *B. amyloliquefaciens* strain and evaluating how it impacts its attraction toward *A. thaliana* and other plant root exudates.

McpA mediates attraction toward glucose and α-methylglucoside by stimulating a rapid tumbling response in the presence of a decreasing gradient of glucose (19, 21). Here, we observed that cells devoid of this chemoreceptor had an increased attraction toward root exudates, which constitutes the first observation of a repellent-like activity mediated via that chemoreceptor. A molecule present in the root exudates could, upon binding to McpA, induce the same conformational change caused by a decreasing glucose concentration. Interestingly, the *mcpA* deletion mutant also displayed increased root colonization compared to WT cells (see Fig. S6 in the supplemental material). This result reinforces the idea that *A. thaliana* secretes a repellent molecule(s), sensed by McpA, in addition to the attractive molecule(s). This observation is particularly interesting from a biocontrol point of view, since making *B. subtilis* insensitive to a putative repellent(s) could improve its root colonization efficiency and its plant growth-promoting (PGP) effect.

While the various chemoreceptors encoded by *B. subtilis* are involved in root colonization, they are not well conserved through strains and species. Yao et al. reported a similar observation for *R. solanacearum*: the two strains GMI1000 and UW551 share only 11 identical chemoreceptors out of 19 and 17, respectively (16). This study also suggested that the different chemotactic response profile of these strains could be attributed to differences in the kind and numbers of chemoreceptors for each strain (16). Plant specificity can also be observed with environmental *Bacillus* spp. strains. Zhang et al. observed that a *B. subtilis* strain isolated from a banana tree rhizosphere and a *B. amyloliquefaciens* isolate from a cucumber plant rhizosphere both showed preferential colonization of their original host (55). Similar to *R. solanacearum*, such behaviors could possibly be attributed to *B. subtilis* strain-specific chemoreceptors endowing them with specificity toward certain plants but not others. This chemoreceptor-mediated plant specificity could also impact biocontrol activity of *B. subtilis*, since we showed here that functional chemotaxis is required for optimal plant protection. Interestingly, Chen et al. demonstrated a strong link between biofilm formation and biocontrol for various *B. subtilis* isolates, but they also observed that certain strains showed poor biocontrol efficiency on tomato plants despite forming robust biofilms in defined media (32). In light of our results, such a lack of effect on the plant could possibly be attributed to a weaker chemotaxis response of these wild *Bacillus* isolates toward tomato root exudates, leading to poor colonization and biocontrol.

A number of chemoreceptors have been involved in the interaction of various bacteria with plant roots. In *R. leguminosarum*, deletion mutants of McpC and of the carbon source sensor McpB are unable to compete with WT cells in nodulation experiments of Trapper peas (11). In *R. pseudosolanacearum*, the malic acid chemoreceptor McpM, but not the amino acid receptor McpA, is involved in mediating virulence (18). A recent study showed that *Pseudomonas putida* cells deleted for either McpU, which mediates chemotaxis toward polyamine, or WspA, which is part of an alternative chemosensory pathway, are much less competitive than WT for maize root colonization (15). Finally, energy taxis via Aer1/2 in *R. solanacearum* and via Tlp1 in *Azospirillum brasilense* appears crucial for optimal root colonization by these bacteria (14, 56). These observations slightly contrast with our results, since we were not successful at defining a clear subset of chemoreceptor proteins required for root colonization. It appears that for *B. subtilis*, several chemoreceptors that are not important for *in vitro* chemotaxis toward root exudates are involved in root colonization. The observations presented here lead us to think that most *B. subtilis* chemoreceptors are involved in root colonization and that they might have overlapping and/or redundant functions. This hypothesis is in agreement with the fact that the rhizosphere is a very favorable niche for *B. subtilis* and that this bacterium readily colonizes plant roots.

## MATERIALS AND METHODS

### Strains, media, and culture conditions.

Strains used in the study are listed in Table S1 in the supplemental material. *B. subtilis* strain NCIB 3610 was used as the wild-type strain, since its capacity to form biofilms is important for plant root colonization experiments longer than 4 h (Fig. 1B and 2B). OI WT and OI *Δ10* strains, obtained from Georges Ordal (University of Illinois), are the only strains with a non-3610 background and were used only for the experiment shown in Fig. 4B. For routine growth, cells were propagated on Luria-Bertani (LB) medium. When necessary, antibiotics were used at the following concentrations: MLS (1 μg ml^−1^ erythromycin, 25 μg ml^−1^ lincomycin), spectinomycin (100 μg ml^−1^), tetracycline (10 μg ml^−1^), chloramphenicol (5 μg ml^−1^), and kanamycin (10 μg ml^−1^). Media used for these experiments were MSN (5 mM potassium phosphate buffer [pH 7], 0.1 M morpholinopropanesulfonic acid [pH 7], 2 mM MgCl_2_, 0.05 mM MnCl_2_, 1 μM ZnCl_2_, 2 μM thiamine, 700 μM CaCl_2_, 0.2% NH_4_Cl) and MSNg (MSN supplemented with 0.05% glycerol, for root colonization).

The Col-0 *A. thaliana* ecotype was used throughout the study (a kind gift from Kamal Bouarab, Université de Sherbrooke). Seeds were surface sterilized with 70% ethanol followed by 0.3% (vol/vol) sodium hypochlorite and germinated on Murashige-Skoog medium (Sigma) with 0.7% agar with 0.05% glucose in a growth chamber at 25°C.

### Strain construction.

The long-flanking homology PCR (LFH-PCR) technique was used to generate deletion strains (57). Primers used for construction of the deletion mutants are listed in Table S2. PCR products for gene deletions were introduced into *B. subtilis* strain PY79 by natural competence (58). Gene deletions were then transferred to strain NCIB3610 or other appropriate mutant strains by SPP1-mediated generalized transduction (59). Of note, *mcpC*::*erm* was constructed by transferring the *mcpC*::*erm* construction from strain OI3280 (kind gift from Georges Ordal) to strain 3610 by SPP1 phage transduction.

### Microscopy.

To view bacteria on the root surfaces, seedlings were examined with a Nikon Eclipse TE2000-U microscope equipped with a 60× Plan Apo oil objective lens, and pictures were taken with a Nikon D7000 digital camera (real-time movie) or with a Hamamatsu digital camera (model ORCA-ER; for fluorescence detection). Figure 1 presents frames from Movie S1 in the supplemental material that were selected with the iMovie software. The fluorescence signal was detected using a CFP/yellow fluorescent protein (YFP) dual-band filter set (catalog no. 52019; Chroma). All images were taken at the same exposure time, processed identically for compared image sets, and prepared for presentation using MetaMorph and Photoshop software. Each image is representative of at least 12 root colonization assays performed in three independent experiments.

### Root exudate preparation.

To collect root exudates, 7-day-old seedlings were transferred to a 24-well plate containing 1 ml of MSN and 12 seedlings per well, and incubated for 1 week in the greenhouse on an orbital shaker at 100 rpm. Plants were then removed, and the contents of the wells were filtered with a 0.22-μm filter.

### Capillary assay.

Capillary assays were performed using a slightly modified protocol from those described by Adler et al. (60) and Ordal (61). One-day-old colonies were inoculated in 3 ml LB, rolled at 37°C for 3 h, and then washed twice in chemotaxis buffer [10 mM potassium phosphate buffer (pH 7.0), 0.1 mM EDTA, 0.05% glycerol, 5 mM sodium-d,l-lactate, 0.14 mM CaCl_2_, and 0.3 mM (NH_4_)_2_SO_4_]. The optical density at 600 nm (OD_600_) was adjusted to 0.002 using chemotaxis buffer, and 200 μl of cells was added in each well of a 96-well plate. A 1-μl capillary containing root exudates was dipped in the bacterial solution for 45 min, after which it was withdrawn and rinsed. The capillary’s content was then squeezed out into buffer and diluted, and the number of bacteria was determined by CFU counting. A 1 mM alanine solution and water were used as positive and negative controls, respectively.

### Colonization assay.

Seven-day-old seedlings were transferred to 300 μl MSNg in a 48-well plate, and medium hosting the plant was inoculated to an OD_600_ of 0.02 with *B. subtilis* (pregrown for 3 h). After 4 h of incubation in the greenhouse, seedlings were harvested and the leaves were cut off. The root was measured, washed, and resuspended in phosphate-buffered saline (PBS; 137 mM NaCl, 2.7 mM KCl, 10 mM Na_2_HPO_4_, 2 mM KH_2_PO_4_). Bacteria were detached from the root by sonication using a Q125 ultrasonic disruptor with a 3-mm probe. The sonicator was set at 30% for 10 1-s pulses, each followed by a 1-s pause. This sonication program did not affect the viability of the cells, since CFU stayed the same before and after sonication. The amount of cells per millimeter of root was then determined by CFU counting. Root length was used instead of root weight, since *A. thaliana* seedlings cannot be weighed accurately due to their small size. Of note, microscopic observations showed that *B. subtilis* was distributed homogeneously on the roots.

### *Pseudomonas syringae* pv. Tomato DC3000 protection assay.

The protection assay with *P. syringae* pv. *tomato* DC3000 was performed as described by Laksmanan et al. (62).

### Statistical analyses.

Statistical analyses were performed using GraphPad Prism 6 software. Comparisons were done using a one-way analysis of variance (ANOVA), followed by Tukey’s multiple-comparison test (set at 5%), except for the experiment illustrated in Fig. 3C, for which the comparison between the 2 bars was done using an unpaired *t* test.

### Bioinformatics.

*B. subtilis* subsp. *subtilis* strain 168 chemoreceptor protein sequences were obtained from NCBI (http://www.ncbi.nlm.nih.gov/nuccore) using GenBank accession numbers NP_388919.1 (HemAT), NP_391002.2 (McpA), NP_391004.2 (McpB), NP_389278.2 (McpC), NP_391003.1 (TlpA), NP_391001.1 (TlpB), NP_388226.2 (TlpC), NP_388617.1 (YfmS), NP_389742.2 (YoaH), and NP_391249.1 (YvaQ). According to Zeigler et al. (63), there is no difference between these sequences and those of NCIB3610. The BLASTN tool (64) was used to find all chemoreceptor ortholog sequences in the annotated protein database of *B. subtilis* UD1022 (CP011534.1), *B. amyloliquefaciens* FZB42 (NC_009725.1), *B. amyloliquefaciens* GB03 (all whole-genome sequence contigs from GCA_000508125.1), *B. methylotrophicus* FKM10 (LNTG00000000), *B. pumilus* INR7 (AYTK00000000) and WP8 (CP010075), and *B. megaterium* Q3 (CP010586.1). An in-house Perl script using the BioPerl toolkit (65) was developed to extract the complete ortholog chemoreceptor sequence from each of the strains based on BLAST analysis with the best high-scoring pairs (HSP). Finally, an alignment of all chemoreceptor protein sequences identified in each *Bacillus* strain was performed using the ClustalW software (66).

## SUPPLEMENTAL MATERIAL

Text S1  Supplementary materials and methods. Download Text S1, PDF file, 0.1 MB

Figure S1  Growth of the motility mutants used in Fig. 2 was monitored based on OD_600_ measurements during incubation in LB at 37°C. Curves show the means of three biological replicates. Download Figure S1, TIF file, 11.2 MB

Figure S2  (A) Pellicle formation of WT and mutant cells in the nonbiofilm-inducing medium MSNc and the biofilm-inducing media MSNc + 0.5% pectin, MSNc + 0.5% xylan, or MSgg (see reference 28 for more details). Images are top-down views of wells and were taken after 24 h and 40 h at 30°C. (B) *B. subtilis* cells were coincubated with *A. thaliana* 1-week old seedlings and imaged at 8 and 16 h postinoculation. Strains used were WT and *hag*, *motA*, and *cheA* mutants, all harboring the biofilm reporter P_*tapA*_-*yfp*. The entire root was imaged at ×63 magnification, and the numbers of fluorescent pixels were counted and then divided by the root’s length (also measured in pixels), allowing quantification of biofilm-forming cells present on the root. For each strain, the bar represents the mean and standard deviation of at least four biological replicates; bars marked with an asterisk indicate results differed significantly from the WT. (C) Representative pictures of WT and *hag*, *motA*, and *cheA* mutants at 8 and 16 h postinoculation. Bars, 20 μm. Download Figure S2, TIF file, 11.2 MB

Figure S3  Attraction of *B. subtilis* toward water, MSN, or *A. thaliana* root exudates in MSN (concentrated or not) was examined in a capillary assay (see Materials and Methods). After incubation, the amount of CFU in the capillaries was evaluated in overnight cultures on LB agar. Root exudates, but not MSN, contained molecules with a specific attraction effect on *B. subtilis*. Download Figure S3, TIF file, 11.2 MB

Figure S4  Attraction of *B. subtilis* WT strain (3610), and *cheA* and *mcpB mcpC tlpC* deletion mutants toward chemotaxis buffer (negative control) or 1% yeast extract. This histogram was obtained from a capillary assay (see Materials and Methods), and bars represent the means of 4 replicates. *cheA* mutant cells were completely defective in chemotaxis toward yeast extract, but both WT and *mcpB mcpC tlpC* mutants cells showed robust attraction. Download Figure S4, TIF file, 11.2 MB

Figure S5  Swimming behavior, measured as the number of tumble events per second, of WT cells and chemotaxis mutants in chemotaxis buffer. The box plot was created using Tukey’s method; results for the mutants cells were not statistically different than results with WT cells. Download Figure S5, TIF file, 11.2 MB

Figure S6  Root colonization assay with various chemoreceptor deletion mutants. One-week-old *A. thaliana* seedlings were coincubated with either WT or mutant *B. subtilis* in MSNg. For the *mcpB-tlpA-mcpA-tlpB yvaQ yoaH yfmS P_hyperspank_-mcpB* strain, 6 μM isopropyl-β-d-thiogalactopyranoside (IPTG) was added to the preculture and the colonization assay medium. This concentration restored WT-like levels of chemoattraction toward asparagine for the mutant strain (data not shown). After 4 h, roots were collected, measured, washed in PBS, and sonicated to disperse the bacteria. CFU were evaluated after overnight culture on LB agar, and numbers are reported relative to the number of CFU per millimeter of root for the WT *B. subtilis* strain. The bars represents the means and standard deviations of five biological replicates; according to a *t* test, the result for *mcpA* differed significantly from that for the WT. Download Figure S6, TIF file, 11.2 MB

Movie S1  Real-time movie of *B. subtilis* WT (3610) cells harboring P_*tapA*_-*yfp* P_*hag*_-*cfp* inoculated with an *A. thaliana* root in MSNg medium at time 0. Download Movie S1, MOV file, 0.8 MB

Table S1  Strains used in this study. Table S1, PDF file, 0.1 MB

Table S2  Primers used for this study. Table S2, PDF file, 0.03 MB
