# Supplementary material for: Bacillus subtilis Early Colonization of Arabidopsis thaliana Roots Involves Multiple Chemotaxis Receptors
Source: mBio. 2016 Nov 29;7(6):e01664-16. doi: 10.1128/mBio.01664-16 (PMC5137498; doi:10.1128/mBio.01664-16)
Supplement: Text S1 — Supplementary materials and methods. Download [file mbo006163083s1.pdf]

Supplementary Material and Methods

Growth curves and biofilm assays

Growth curves were performed as describe in (1), and biofilm assays were performed as describe in (2).

Microscopy and Image analysis

Images for root colonization (supplementary figure S2) and for swimming behavior (supplementary figure S5) were taken using a Zeiss AxioObserver Z1 equipped with a Zeiss Axiocam 506 mono. A 63X/1.4 Plan Apo oil objective was used for the root colonization experiment and the YFP was detected using a bandpass filter set (Zeiss filter set 46). Images pre-processing, segmentation and analysis were performed using the software CellProfiler (cellprofiler.org) (2). To measure the tumbling rate of the cells, 10 seconds time lapse were made at 35 frames per second using a 20x/0.3 phase contrast objective. The trajectories of the cells were found and characterized using the TrackMate plugin in Fiji (Fiji.sc) (3). For each strain, 20 bacteria trajectories of at least 5 seconds were examined and the number of tumbling event were counted.

(1) Chai Y, Beauregard PB, Vlamakis H, Losick R, Kolter R. (2012) Galactose metabolism plays a crucial role in biofilm formation by *Bacillus subtilis*, *mBio* 3(4)

(2) Beauregard PB, Chai Y, Vlamakis H, Losick R and Kolter R. (2013) *Bacillus subtilis* biofilm induction by plant polysaccharides, *Proc. Natl. Acad. Sci. USA* 110(17):E1621-30

(3) Lamprecht, M. R., Sabatini, D. M., & Carpenter, A. E. (2007). CellProfiler: Free, versatile software for automated biological image analysis. *BioTechniques*, *42*(1), 71–75.

(4) Schindelin, J., Arganda-Carreras, I., Frise, E., Kaynig, V., Longair, M., Pietzsch, T., … Cardona, A. (2012). Fiji: an open-source platform for biological-image analysis. *Nature Methods*, *9*(7), 676–682.
