# Supplementary figures and images for: Bacillus subtilis Early Colonization of Arabidopsis thaliana Roots Involves Multiple Chemotaxis Receptors
Source: mBio. 2016 Nov 29;7(6):e01664-16. doi: 10.1128/mBio.01664-16 (PMC5137498; doi:10.1128/mBio.01664-16)

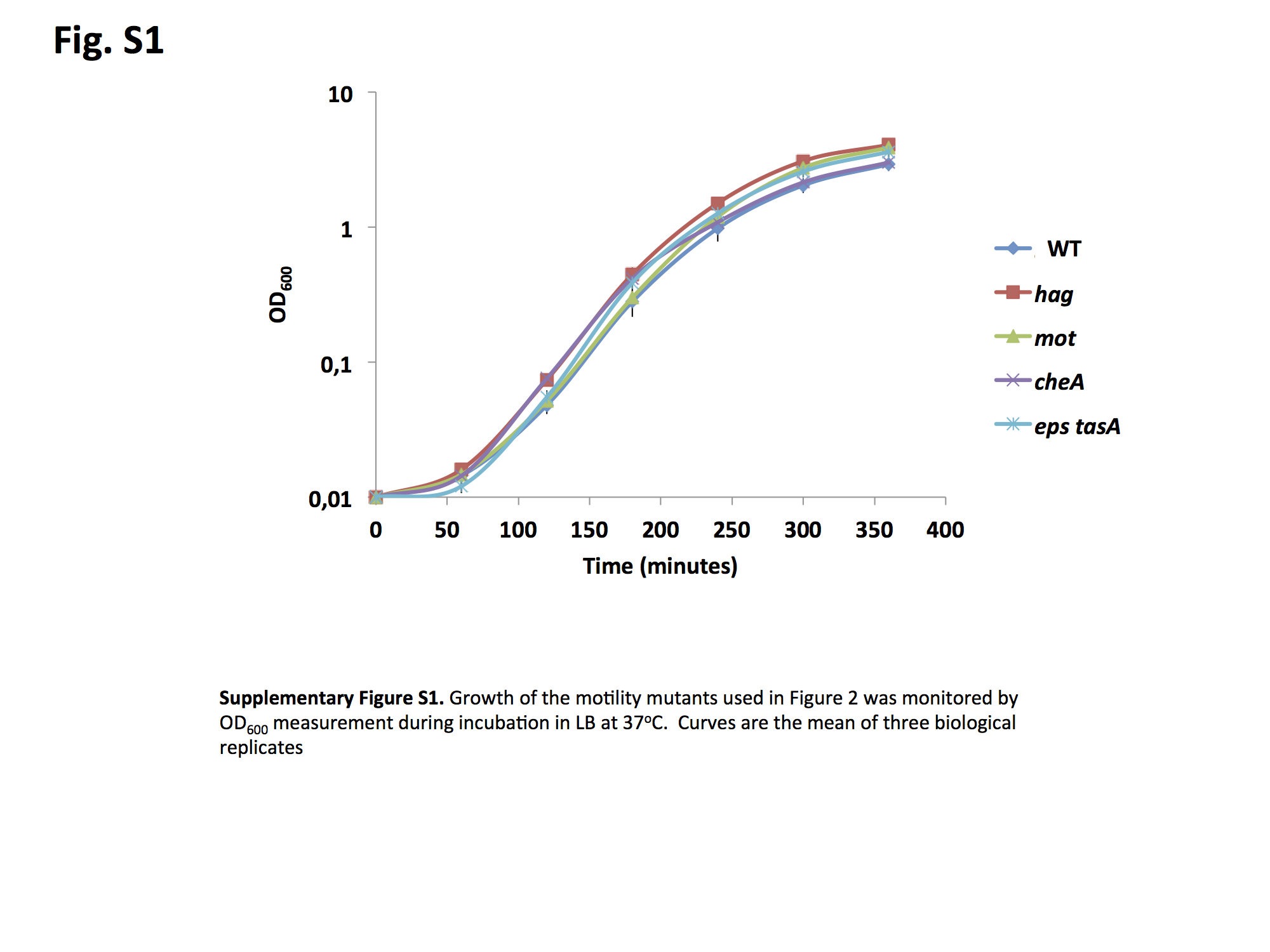

Supplement: Figure S1 — Growth of the motility mutants used in Fig. 2 was monitored based on OD600 measurements during incubation in LB at 37°C. Curves show the means of three biological replicates. Download [file mbo006163083sf1.tif]

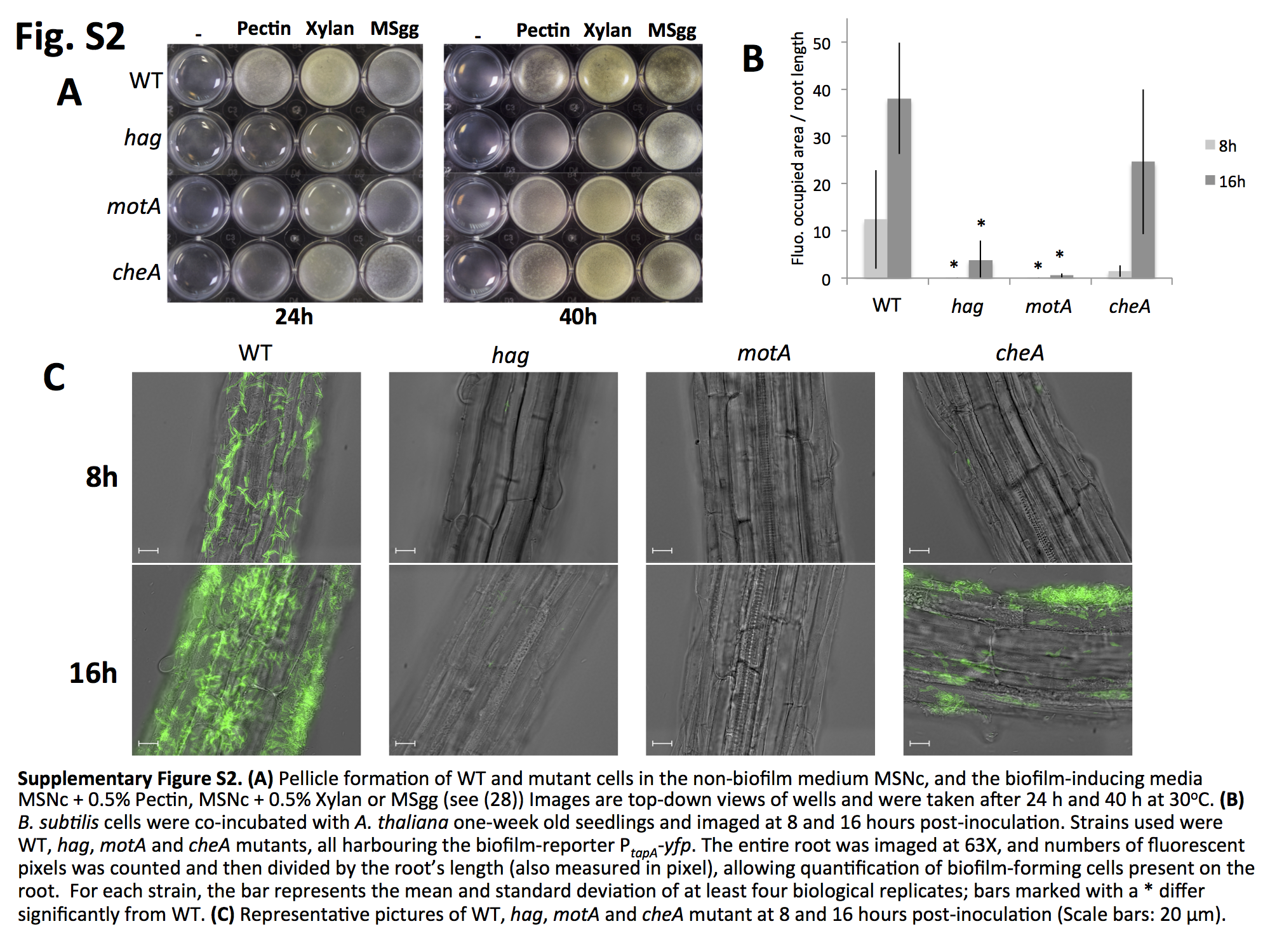

Supplement: Figure S2 — (A) Pellicle formation of WT and mutant cells in the nonbiofilm-inducing medium MSNc and the biofilm-inducing media MSNc + 0.5% pectin, MSNc + 0.5% xylan, or MSgg (see reference 28 for more details). Images are top-down views of wells and were taken after 24 h and 40 h at 30°C. (B) B. subtilis cells were coincubated with A. thaliana 1-week old seedlings and imaged at 8 and 16 h postinoculation. Strains used were WT and hag, motA, and cheA mutants, all harboring the biofilm reporter PtapA-yfp. The entire root was imaged at ×63 magnification, and the numbers of fluorescent pixels were counted and then divided by the root’s length (also measured in pixels), allowing quantification of biofilm-forming cells present on the root. For each strain, the bar represents the mean and standard deviation of at least four biological replicates; bars marked with an asterisk indicate results differed significantly from the WT. (C) Representative pictures of WT and hag, motA, and cheA mutants at 8 and 16 h postinoculation. Bars, 20 μm. Download [file mbo006163083sf2.tif]

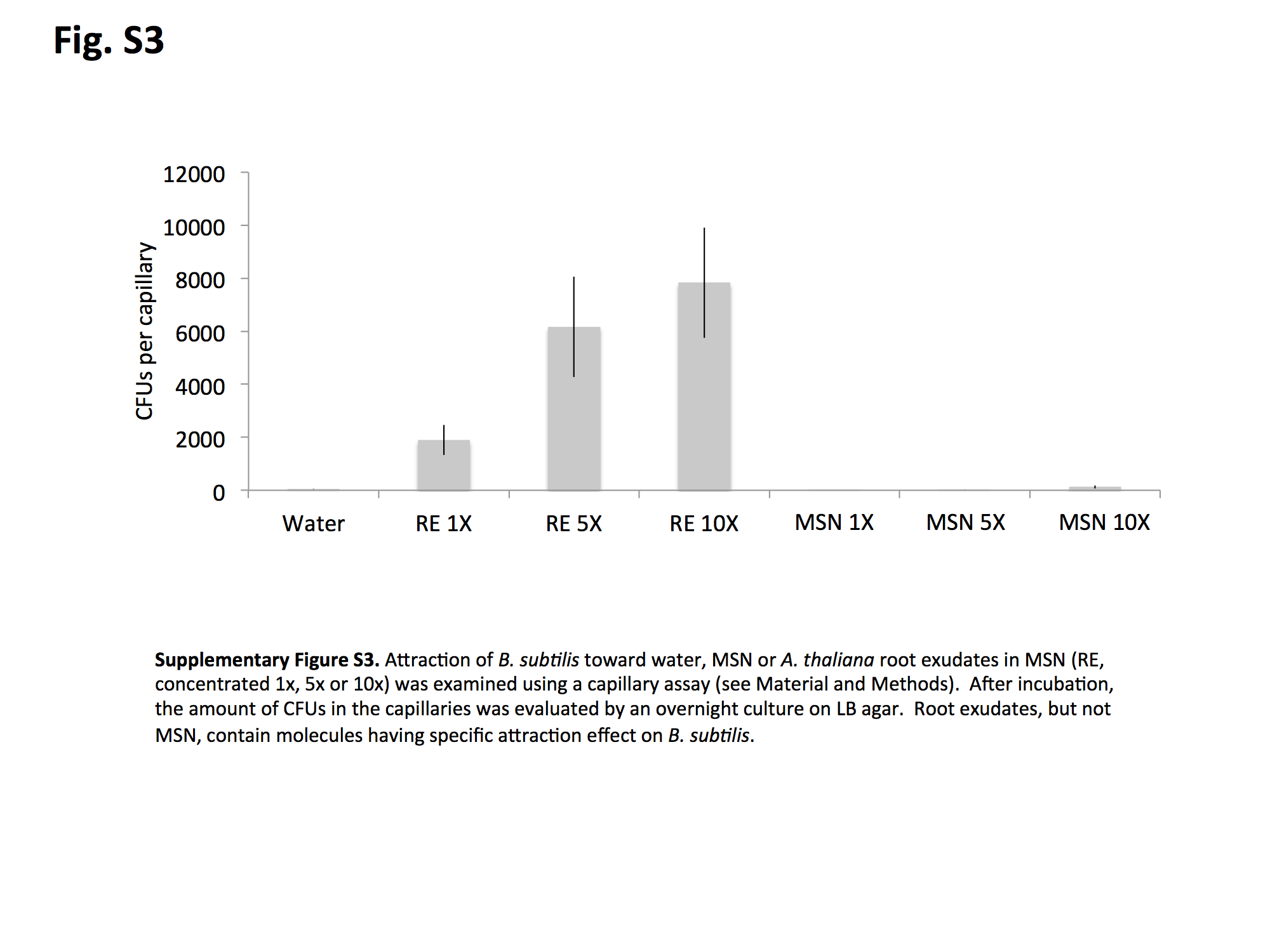

Supplement: Figure S3 — Attraction of B. subtilis toward water, MSN, or A. thaliana root exudates in MSN (concentrated or not) was examined in a capillary assay (see Materials and Methods). After incubation, the amount of CFU in the capillaries was evaluated in overnight cultures on LB agar. Root exudates, but not MSN, contained molecules with a specific attraction effect on B. subtilis. Download [file mbo006163083sf3.tif]

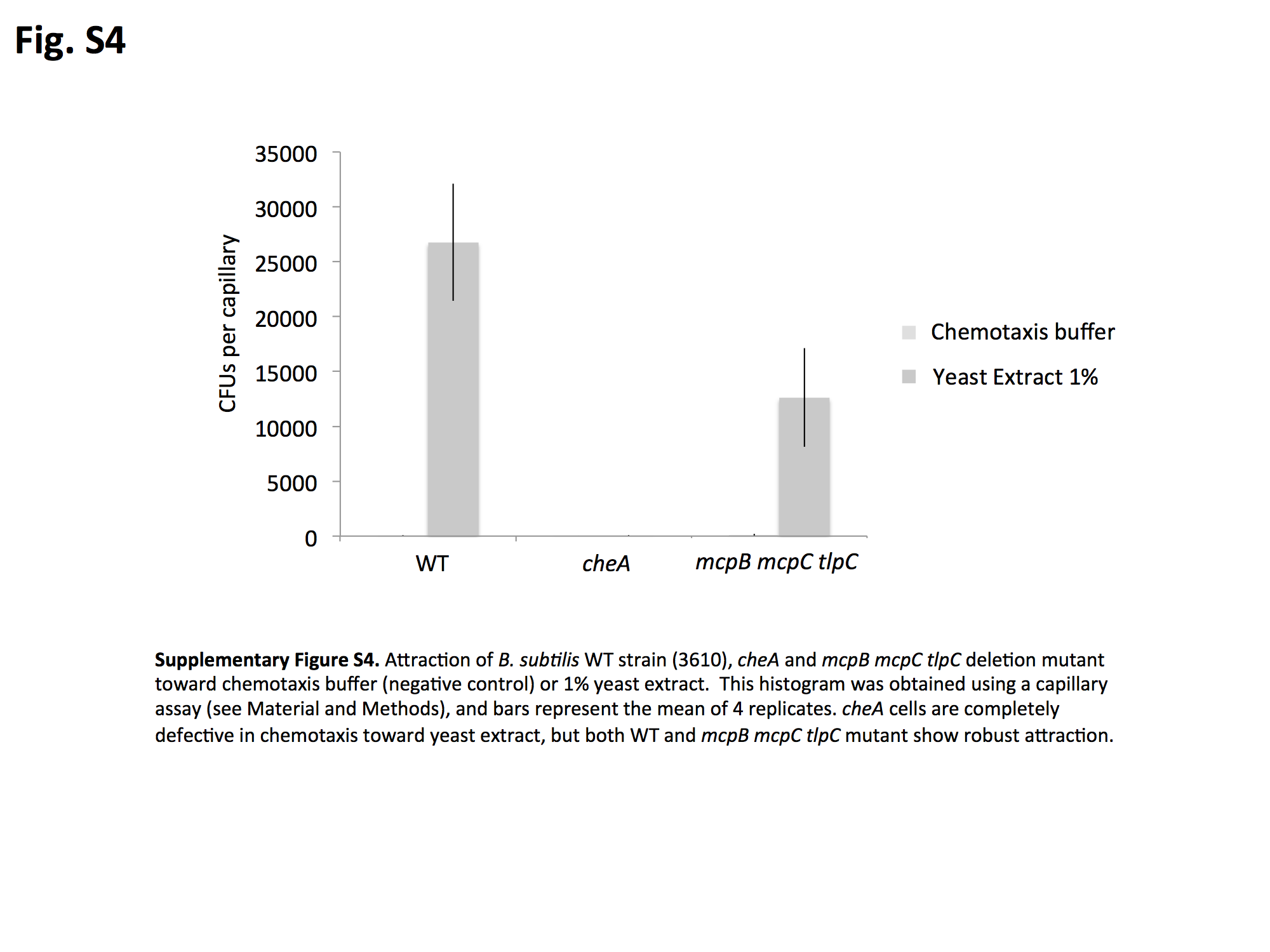

Supplement: Figure S4 — Attraction of B. subtilis WT strain (3610), and cheA and mcpB mcpC tlpC deletion mutants toward chemotaxis buffer (negative control) or 1% yeast extract. This histogram was obtained from a capillary assay (see Materials and Methods), and bars represent the means of 4 replicates. cheA mutant cells were completely defective in chemotaxis toward yeast extract, but both WT and mcpB mcpC tlpC mutants cells showed robust attraction. Download [file mbo006163083sf4.tif]

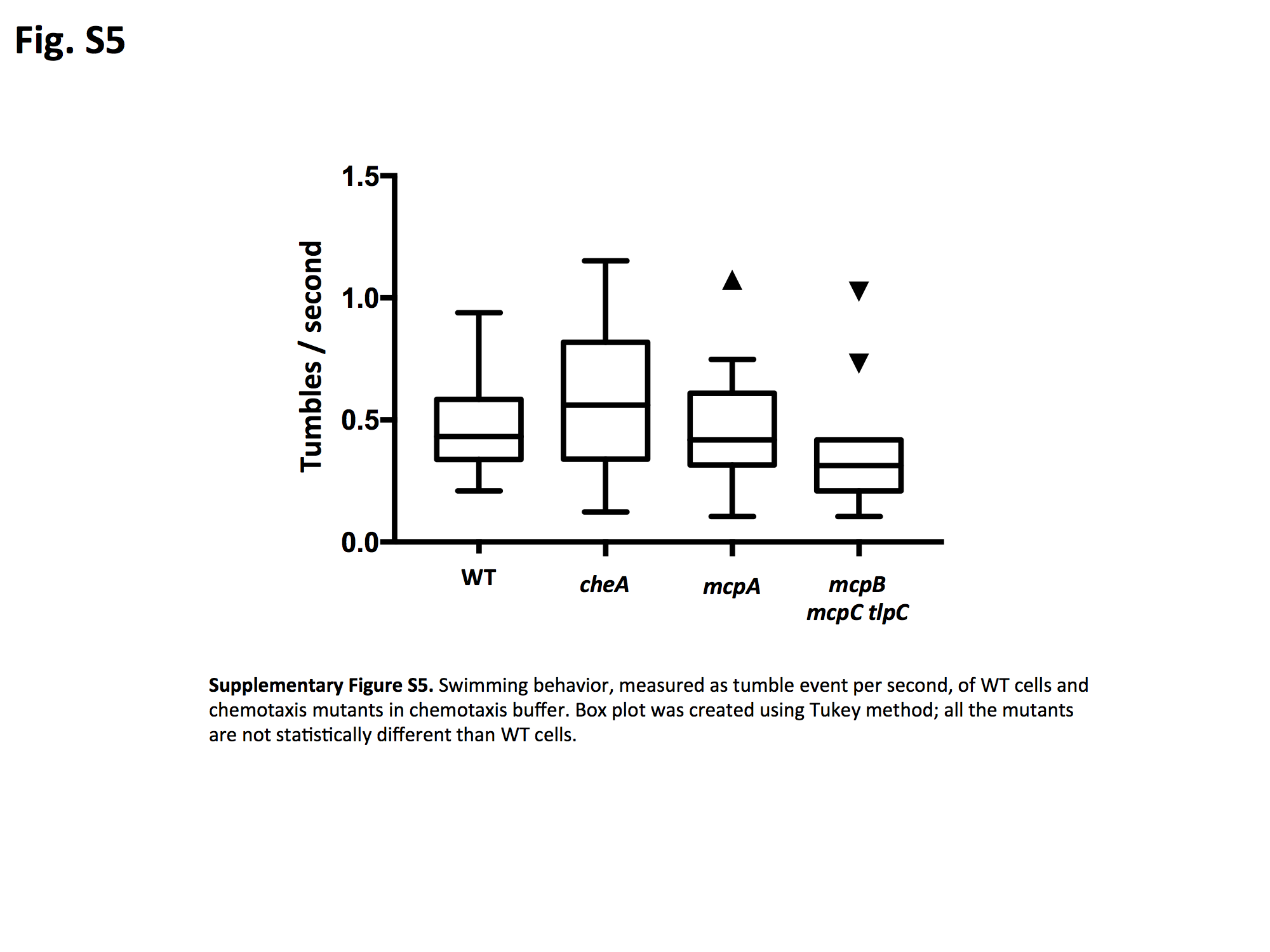

Supplement: Figure S5 — Swimming behavior, measured as the number of tumble events per second, of WT cells and chemotaxis mutants in chemotaxis buffer. The box plot was created using Tukey’s method; results for the mutants cells were not statistically different than results with WT cells. Download [file mbo006163083sf5.tif]

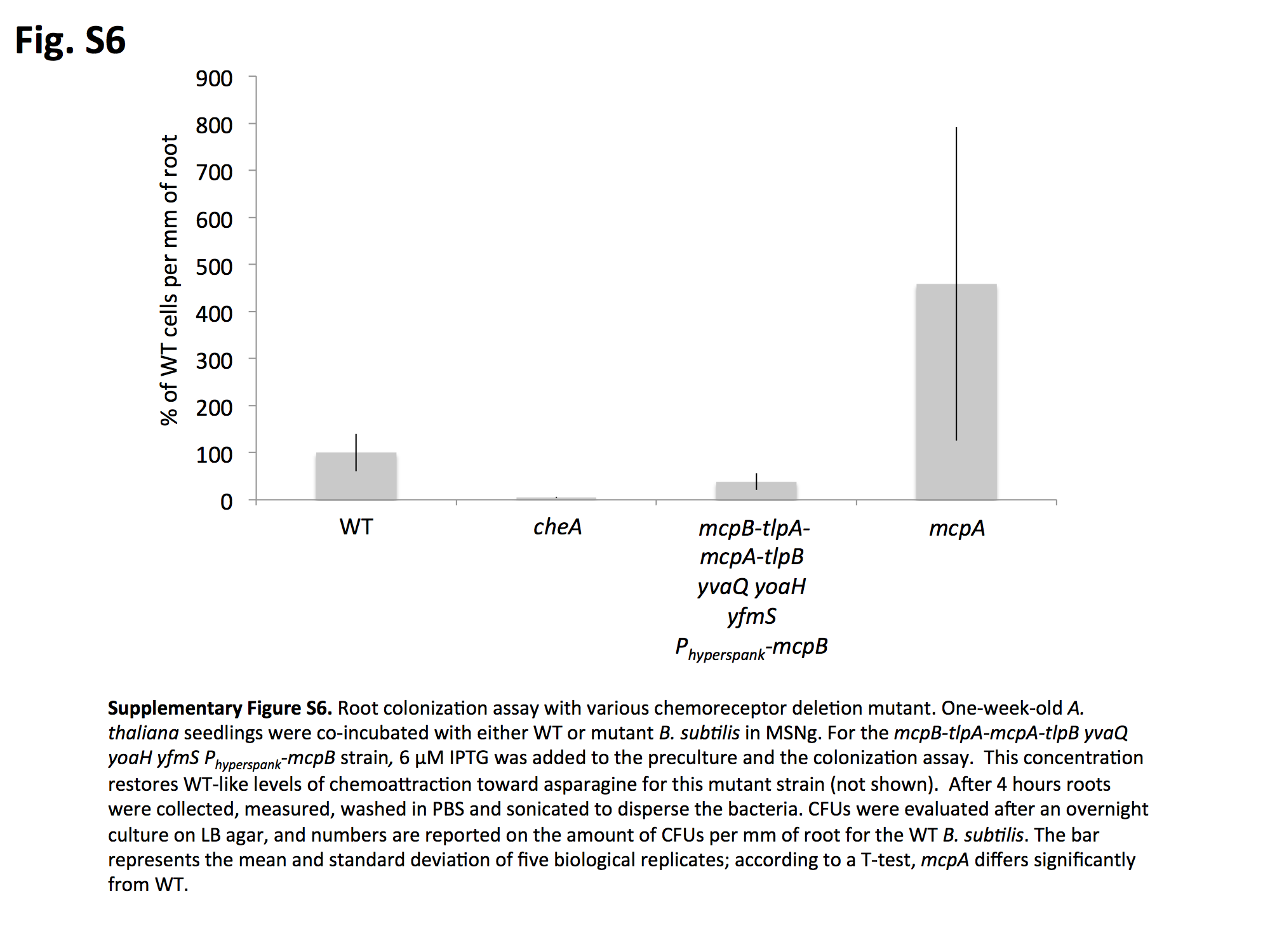

Supplement: Figure S6 — Root colonization assay with various chemoreceptor deletion mutants. One-week-old A. thaliana seedlings were coincubated with either WT or mutant B. subtilis in MSNg. For the mcpB-tlpA-mcpA-tlpB yvaQ yoaH yfmS Phyperspank-mcpB strain, 6 μM isopropyl-β-d-thiogalactopyranoside (IPTG) was added to the preculture and the colonization assay medium. This concentration restored WT-like levels of chemoattraction toward asparagine for the mutant strain (data not shown). After 4 h, roots were collected, measured, washed in PBS, and sonicated to disperse the bacteria. CFU were evaluated after overnight culture on LB agar, and numbers are reported relative to the number of CFU per millimeter of root for the WT B. subtilis strain. The bars represents the means and standard deviations of five biological replicates; according to a t test, the result for mcpA differed significantly from that for the WT. Download [file mbo006163083sf6.tif]
