# Supplementary material for: Bacillus subtilis Early Colonization of Arabidopsis thaliana Roots Involves Multiple Chemotaxis Receptors
Source: mBio. 2016 Nov 29;7(6):e01664-16. doi: 10.1128/mBio.01664-16 (PMC5137498; doi:10.1128/mBio.01664-16)
Supplement: Table S1 — Strains used in this study. [file mbo006163083st1.pdf]

Table S1. Strains use in this study

Strain	Genotype	Reference/source
NCIB3610	**Wild type**. Undomesticated strain	Kolter lab stock
HV1048	*amyE*::P_{hag}-*cfp* (spec)	(1)
DL821	*lacA*::P_{tapA}-*yfp* (erm)	(2)
PB293	*amyE*::P_{spac}-*cfp* (spec)	This study
HV1150	*hag::tet*	(1)
PB371	*motA::kan*	This study
RL2666	*cheA::tet*	(3)
HV1235	*eps::tet tasA::kan*	(4)
PB408	*mcpA::tet*	This study
PB409	*mcpB::tet*	This study
PB410	*tlpA::tet*	This study
PB411	*tlpB::tet*	This study
PB418	*mcpC::erm*	This study
PB451	*tlpC::spc*	This study
PB455	*mcpB::tet mcpC::mls tlpC::spc*	This study
PB486	*yvaQ::cm*	This study
ZK4262	*hemAT::erm*	Roberto Kolter
PB434	*yfmS::kan*	This study
PB465	*yoaH::kan*	This study
PB461	*mcpB::tet mcpC::mls tlpC::spc hemAT::kan*	This study
OI1085	*his- met- trp-* (OI WT)	(5)
OI3545	*(mcpA mcpB tlpA tlpB)101::cat mcpC4::erm tlpC::cat hemAT::erm yfmS::erm yoaH::erm yvaQ::erm* (OI *Δ10*)	(5)

*Unless indicated, strains are derivatives of *B. subtilis* 3610. Antibiotics: spectinomycin (spec), kanamycin (kan), erm (mls), chloramphenicol (cm), tetracycline (tet).

1. **Vlamakis H, Aguilar C, Losick R, Kolter R**. 2008. Control of cell fate by the formation of an architecturally complex bacterial community. Genes Dev **22**:945–53.

2. **López D, Vlamakis H, Losick R, Kolter R**. 2009. Paracrine signaling in a bacterium. Genes Dev **23**:1631–1638.

3. **Kearns DB, Losick R**. 2004. Swarming motility in undomesticated *Bacillus subtilis*. Mol Microbiol **49**:581–590.

4. **Aguilar C, Vlamakis H, Guzman A, Losick R, Kolter R**. 2010. KinD is a checkpoint protein linking spore formation to extracellular-matrix production in *Bacillus subtilis* biofilms. MBio **1**.

5. **Hou S, Larsen RW, Boudko D, Riley CW, Karatan E, Zimmer M, Ordal GW, Alam M**. 2000. Myoglobin-like aerotaxis transducers in Archaea and Bacteria. Nature **403**:540–4.
