# Supplementary material for: Bacillus subtilis Early Colonization of Arabidopsis thaliana Roots Involves Multiple Chemotaxis Receptors
Source: mBio. 2016 Nov 29;7(6):e01664-16. doi: 10.1128/mBio.01664-16 (PMC5137498; doi:10.1128/mBio.01664-16)
Supplement: Table S2 — Primers used for this study. [file mbo006163083st2.pdf]

Table S2. Primers use in this study

| Primer name | Sequence |
|---|---|
| motA_up_fwd | 5'-ggagaacttgaagaaacattggcgg |
| motA_up_rev | 5'-cctatcacctcaaatggttcgctggacgctcaatgccacaaaagcaaga |
| motA_down_fwd | 5'-cgagcgcctacgaggaatttgtatcggcagaacaaggagaggcgcaaaatg |
| motA_down_rev | 5'-cgcctctcggcaaaatgagaacttc |
| mcpA_up_fwd | 5'-caagtggtgaaagggctgg |
| mcpA_up_rev | 5'-gagaacaacctgcaccattgcaaga gatctttgttttatgagttggag |
| mcpA_down_fwd | 5'-gggatcaactttgggagagagttc cgggatatgaccaaacgatt |
| mcpA_down_rev | 5'-agccttcttttccgattttga |
| mcpB_up_fwd | 5'-cggatcaaattcaggattctta |
| mcpB_up_rev | 5'-gagaacaacctgcaccattgcaaga cttcagccaattgataaaagtc |
| mcpB_down_fwd | 5'-gggatcaactttgggagagagttc gcgcagatggcagaagagc |
| mcpB_down_rev | 5'-gacaattgttttgcctttattgg |
| tlpA_up_fwd | 5'-caacggaatcgctgaccaaac |
| tlpA_up_rev | 5'-gagaacaacctgcaccattgcaaga gatctgcgaatagtggtgagt |
| tlpA_down_fwd | 5'-gggatcaactttgggagagagttc ctgacgcttgaaagaatgtctg |
| tlpA_down_rev | 5'-ctgaaccatcttttgtctgctg |
| tlpB_up_fwd | 5'-gttaaagaagcggagcaagtc |
| tlpB_up_rev | 5'-gagaacaacctgcaccattgcaaga gctgtttgatccattgtatgaat |
| tlpB_down_fwd | 5'-gggatcaactttgggagagagttc gaagaacttcaggacattacg |
| tlpB_down_rev | 5'-cattaatacaagctcggcgtg |
| tlpC_up_fwd | 5'-agaagtgatccagcaaaagcctgac |
| tlpC_up_rev | 5'-cttgataataagggtaactattgcctaagcatcacagtgccaactgatgc |
| tlpC_down_fwd | 5'-gggtaactagcctcgccggtccacgaggagcttaccggtatcataagcca |
| tlpC_down_rev | 5'-tttcgctgtgcctcaaaggaaatgg |
| yvaQ_up_fwd | 5'-atccgcgaagcatctgaaggctatc |
| yvaQ_up_rev | 5'-cttgataataagggtaactattgcccaggctgaatttgcgggaaatcgtc |
| yvaQ_down_fwd | 5'-gggtaactagcctcgccggtccacgtgaagagcttgagcagctggccaat |
| yvaQ_down_rev | 5'-gggaagaggtcaatgaacgtgcttg |
| yfmS_up_fwd | 5'-gcggatcatctcatttaccggttct |
| yfmS_up_rev | 5'-cctatcacctcaaatggttcgctgttttggccggtatattcacgagcag |
| yfmS_down_fwd | 5'-cgagcgcctacgaggaatttgtatcgttgcgaaaatggccgaaaaagcgct |
| yfmS_down_rev | 5'-gaaatatctatcgtgcgtcaccagc |
| yoaH_up_fwd | 5'-catgtaatccggacttctccccatc |
| yoaH_up_rev | 5'-cctatcacctcaaatggttcgctgcatgtgtcgttttccctttccgcac |
| yoaH_down_fwd | 5'-cgagcctacgaggaatttgtatcggggaaatctcctatgccgcagaatc |
| yoaH_down_rev | 5'-gcatgaatacctgcccaacagacag |
| hemAT_up_fwd | 5'-tccacccaagatctagtgacgatcg |
| hemAT_up_rev | 5'-cctatcacctcaaatggttcgctgctcagcatctcccaacctgaccatt |
| hemAT_down_fwd | 5'-cgagcgcctacgaggaatttgtatcgccgttgattcgcttgtcatcctgac |
| hemAT_down_rev | 5'-gggcgatgtttcggcagtagatgaa |
